# Optimizing the Transition of Care for Postpartum Preeclampsia: A Scoping Review of Management Strategies and Missed Opportunities

**DOI:** 10.7759/cureus.108253

**Published:** 2026-05-04

**Authors:** Nadine Z Chrestay, Noah O Chrestay, Monique Brotman

**Affiliations:** 1 Obstetrics and Gynecology, Family Medicine, St. George's University, True Blue, GRD; 2 Department of Obstetrics/Gynecology, West Suburban Medical Center, Oak Park, USA

**Keywords:** eclampsia, healthcare equity, maternal morbidity, postpartum care, postpartum preeclampsia, remote monitoring, scoping review, transition of care

## Abstract

Hypertensive disorders of pregnancy remain a primary driver of preventable maternal morbidity, specifically during the high-risk transition from hospital discharge to long-term primary care. This scoping review maps the "transition gap" within the fourth trimester to determine why traditional management strategies often fail to protect patients at risk for late-onset postpartum preeclampsia. Following Preferred Reporting Items for Systematic reviews and Meta-Analyses extension for Scoping Reviews (PRISMA-ScR) guidelines, a systematic search of PubMed, the Cochrane Library, and Google Scholar was conducted for peer-reviewed literature published between January 2010 and December 2025. A two-stage screening process involving independent reviewers led to the inclusion of 52 studies from an initial identification of 14,577 records. Findings indicate that conventional office-based follow-up models suffer from significant patient attrition and frequently overlook "masked" hypertension or de novo blood pressure spikes occurring after the initial discharge window. While remote patient monitoring (RPM) shows high feasibility for maintaining engagement, its clinical success is often hampered by structural and systemic barriers, including racial disparities, neighborhood-level socioeconomic disadvantage, and the loss of insurance coverage in the late postpartum period. To optimize outcomes, healthcare systems must transition toward specialized multidisciplinary transition clinics and standardized pharmacological protocols. By leveraging extended Medicaid coverage, integrating RPM technology, and adopting more stringent blood pressure targets of <130/80 mmHg, clinicians can bridge current fragmentation and transform the postpartum period into a vital window for lifelong cardiovascular health optimization.

## Introduction and background

Hypertensive disorders of pregnancy (HDP) remain one of the leading causes of preventable maternal morbidity and mortality, but clinically significant complications can occur after delivery, not only antepartum. Postpartum preeclampsia is increasingly recognized as an important contributor to emergency department visits and hospital readmissions in the weeks following birth. Clinically, the condition is defined as a systemic vasospastic disorder characterized by new-onset hypertension, typically a systolic blood pressure ≥140 mmHg or diastolic ≥90 mmHg, occurring after delivery, often in association with end-organ dysfunction such as proteinuria, renal insufficiency, or hepatic involvement. The clinical presentation is frequently insidious; symptoms such as persistent, worsening headache, visual disturbances, or epigastric pain are often mistakenly attributed by patients to the general discomfort or exhaustion of the early postpartum period. The severity of this condition stems from its potential for rapid progression into life-threatening eclamptic seizures, HELLP syndrome (hemolysis, elevated liver enzymes, and low platelet count), and acute cerebrovascular accidents. Population-level analyses suggest that a substantial share of severe outcomes and deaths related to hypertensive disorders actually occur in the early postpartum window, highlighting the need for vigilant post-discharge surveillance and timely intervention [1].

Evidence-based approaches exist to reduce severe morbidity among patients at risk for postpartum preeclampsia and subsequent eclampsia. These approaches include consistent blood pressure monitoring both at home and in the clinic, patient education on red flags, and when to initiate treatment [2]. Clinical guidelines from American College of Obstetricians and Gynecologists (ACOG) emphasize early postpartum blood pressure assessment for patients with HDP, recommending an initial evaluation within seven to 10 days postpartum, and as early as 72 hours for those with severe features [3]. This professional standard accounts for the clinical reality that postpartum blood pressure typically peaks between days three and five after delivery. Because this physiological window often coincides with the period immediately following hospital discharge, patients are at their most vulnerable for acute deterioration during this transition, highlighting the critical need for the structured surveillance protocols identified in this review. Emerging care models, such as remote blood pressure monitoring programs at home, have demonstrated improved blood pressure ascertainment compared with traditional office-based follow-up. This shows that prevention is possible when systems help patients monitor themselves, enabling a faster clinical response when issues arise [4-6].

Despite these strategies and guidance, postpartum preeclampsia and severe postpartum hypertension continue to occur. The persistence of severe outcomes appears to reflect improper implementation and continuity gaps rather than a lack of evidence-based recommendations. Reported contributors include delayed recognition of postpartum hypertension, particularly for de novo cases, inconsistent scheduling or completion of early follow-up, and limited patient understanding of warning symptoms. This fragmentation is further exacerbated by the inadequate transition between inpatient obstetrics and primary care, a critical window, as HDP are now recognized as early markers for long-term cardiovascular disease (CVD). Disparities in access and follow-up reliability further compound risk; while targeted remote monitoring programs have been studied as a strategy to reduce these gaps, recent meta-analytical evidence suggests that such models are not superior to standard care in preventing hypertensive-related readmissions and may significantly increase emergency department utilization due to the lack of patient education [7]. Collectively, these issues highlight the need to synthesize not only preventive strategies but also the specific points of failure in follow-up that allow the stable postpartum hypertension to severe morbidity or eclampsia [5,8].

Postpartum hypertension may represent the initial presentation of what later can manifest as preeclampsia. Accordingly, it is considered within the at-risk clinical pathway in this review. The objective of this scoping review is to map postpartum management strategies intended to optimize the transition of care for postpartum preeclampsia and identify the patient-, provider-, and system-level barriers that contribute to missed opportunities for prevention after delivery.

## Review

Methods

*Review Design* 

We followed the Preferred Reporting Items for Systematic Reviews and Meta-Analyses extension for Scoping Reviews (PRISMA-ScR) guidelines [9] to ensure our process was transparent and reproducible.

Eligibility Criteria and Study Selection

To ensure a focused analysis on the transition from acute to longitudinal care, this review included studies involving postpartum individuals diagnosed with preeclampsia, including those with severe features, or other HDP. For the purposes of this review, the postpartum period was defined as the window from 48 hours through six weeks after delivery. Literature was eligible for inclusion if it explicitly outlined postpartum follow-up protocols, longitudinal monitoring strategies, or interventions designed to mitigate the risk of hospital readmission, such as remote patient monitoring (RPM) and specialized transition clinics. Included studies were further required to report on specific clinical outcomes, including eclamptic complications or emergency department utilization. The search was limited to English-language, peer-reviewed articles published between January 2010 and March 2026. Regarding the immediate postpartum period, studies focusing on the initial 48 hours were only retained if they successfully tracked outpatient progress and the transition to home care. While case reports and case series were generally excluded due to limited sample sizes, select cases were retained where they established the clinical rationale for specific transition-of-care models or described novel clinical presentations. Conversely, editorials, commentaries, and non-human studies were excluded to maintain the integrity of the primary data synthesis.

*Information Sources and Search Strategy* 

We searched PubMed/MEDLINE, Google Scholar, and The Cochrane Library to capture an extensive range of evidence across clinical obstetrics and health systems research. Our strategy primarily targeted two key concepts: (1) the condition (e.g., postpartum preeclampsia or eclampsia) and (2) the timing (e.g., the postpartum or postnatal period). We utilized MeSH terms and Boolean operators to maximize sensitivity and capture both peer-reviewed and gray literature. Full search strings for all databases are documented in Table 1.

**Table 1:** Search strings and Boolean logic This table outlines the comprehensive electronic search strings, Boolean logic, and MeSH terms utilized across three primary databases: PubMed/MEDLINE, The Cochrane Library, and Google Scholar. The search was restricted to English-language papers published between January 2010 and March 2026. The "Yield" column represents the raw number of results identified in each database prior to the deduplication process performed in Zotero. MeSH, Medical Subject Headings; N, total number of identified records; AND/OR, Boolean operators used to combine search concepts.

Database	Search Strategy (Keywords & Boolean Operators)
PubMed	((preeclampsia[MeSH] OR eclampsia[MeSH] OR "postpartum preeclampsia" OR "postpartum eclampsia")) AND (postpartum[MeSH] OR "after delivery" OR postnatal OR "after birth")
Cochrane	#1: MeSH descriptor: [Pre-Eclampsia] #2: MeSH descriptor: [Eclampsia] #3: postpartum preeclampsia #4: postpartum eclampsia Combined: #1 OR #2 OR #3 OR #4
Google Scholar	"postpartum preeclampsia management protocol"

For example, our final PubMed string was: ((preeclampsia[MeSH] OR eclampsia[MeSH] OR "postpartum preeclampsia" OR "postpartum eclampsia")) AND (postpartum[MeSH] OR "after delivery" OR postnatal OR "after birth"). Our systematic search strategy and database yields are detailed in Table 2.

**Table 2:** Systematic database search strategy and record yields This table summarizes the search architecture, including the databases utilized, the conceptual framework for the search strings, and the resulting record counts prior to duplicate removal. n: Number of individual records identified. MeSH: Medical Subject Headings (official controlled vocabulary for PubMed and Cochrane). Google Scholar: Supplemental source used to capture gray literature and management protocols; the yield reflects relevant records identified to satisfy the total starting count for the scoping review.

Database	Search Detail / Strategy	Date of Search	Results (n)
PubMed/MEDLINE	MeSH and keywords for condition and postpartum timing.	March 23, 2026	2,582
Cochrane Library	MeSH descriptors and keywords for condition variants.	March 23, 2026	2,188
Google Scholar	"Postpartum preeclampsia management" (Filtered for 2016–2026).	March 23, 2026	9,807
Total Identified			14,577

Study Selection

The selection process followed the PRISMA-ScR guidelines [9]. Following the identification of 14,577 records, duplicates (n=11,097) were removed using Zotero reference management software. Screening was then conducted in two distinct stages.

First, titles and abstracts of the remaining 3,480 records were screened for relevance, resulting in the exclusion of 3,271 articles that did not meet the predefined clinical or temporal scope. Second, a full-text eligibility assessment was performed on the remaining 209 studies. During this phase, 157 articles were excluded based on specific criteria, including non-eligible study designs, inappropriate populations (e.g., non-postpartum), or lack of management outcomes.

Ultimately, 52 studies met all the inclusion criteria. Discrepancies at all stages were resolved through consensus-based discussion. The complete attrition process and final record yields are given in the sections below.

Data Extraction

A standardized extraction form was utilized to ensure consistency across the 52 included studies, capturing primary characteristics such as author, year of publication, and sample size. The extraction process identified the various clinical settings involved, including emergency departments, telehealth platforms, and primary care environments, while specifically documenting the timing of postpartum interventions at the 72-hour, seven to 10-day, and six-week milestones. Furthermore, barriers to care were analyzed and categorized across three distinct tiers: patient-level factors such as adherence and transportation; provider-level challenges including risk recognition and adherence to clinical guidelines; and system-level hurdles such as scheduling difficulties, insurance gaps, and fragmented team communication.

Data Synthesis

Following extraction, the data were synthesized thematically to identify overarching patterns in follow-up success and longitudinal management. This synthesis was designed to pinpoint specific clinical and systemic 'points of failure' where the progression from hypertension to life-threatening eclamptic events could be effectively interrupted. By organizing the evidence in this narrative fashion, the review highlights the critical gaps in the transition of care that must be addressed to improve maternal outcomes and bridge the current fragmentation in postpartum surveillance

Results

Study Selection

**Figure 1:**
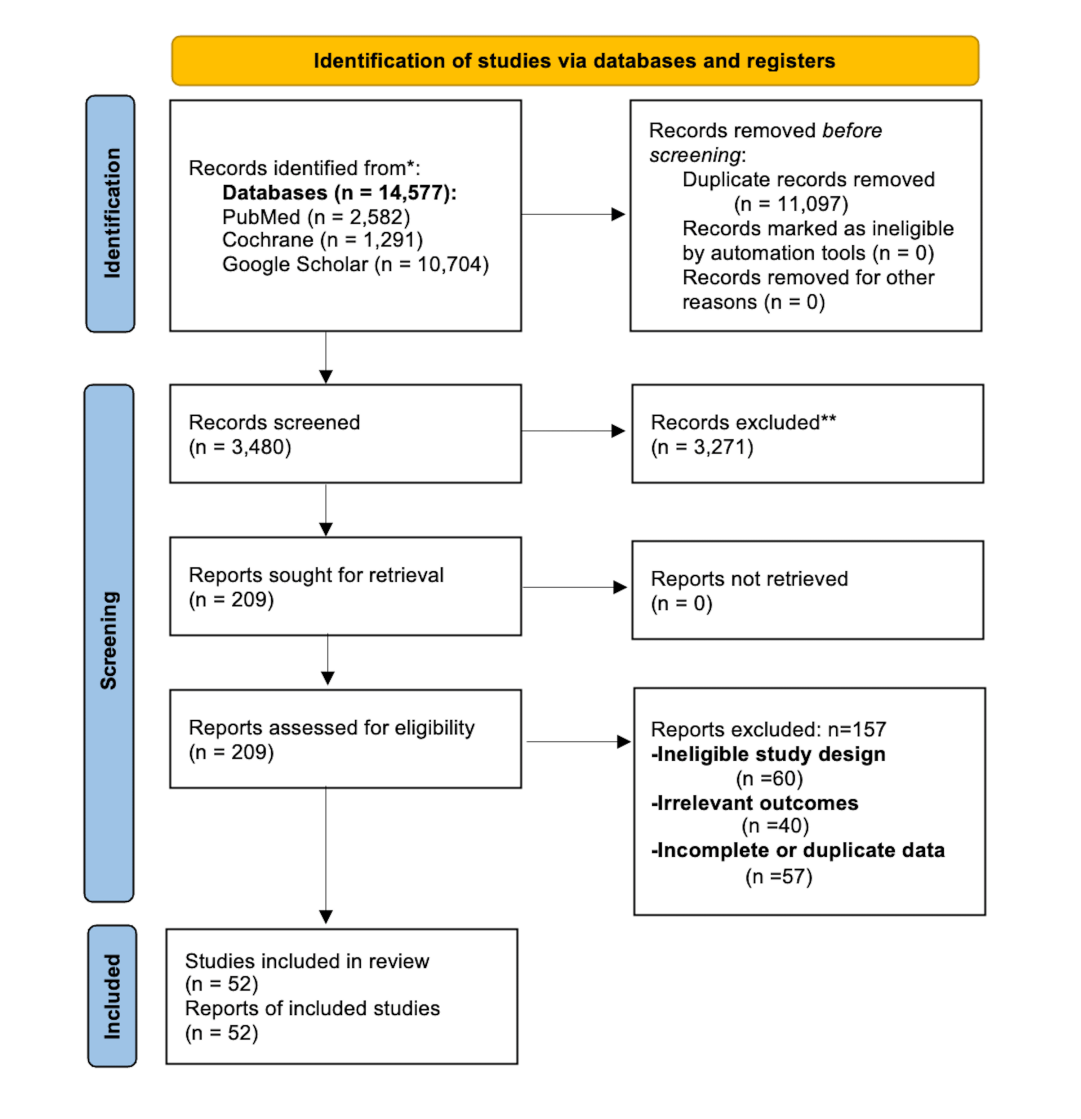
PRISMA flow diagram of the study identification and selection process This Preferred Reporting Items for Systematic Reviews and Meta-Analyses (PRISMA) flow diagram illustrates the systematic identification, screening, and inclusion of studies. The selection process for the 52 included studies was conducted in accordance with the Preferred Reporting Items for Systematic reviews and Meta-Analyses extension for Scoping Reviews (PRISMA-ScR) guidelines [9] using the updated PRISMA 2020 standardized template [10]. n: The number of individual records or studies identified at each stage; Records identified from*: Refers to the total count of unique citations retrieved across all searched electronic databases; Records excluded**: Refers to citations removed during the title and abstract screening phase by human reviewers based on pre-defined inclusion/exclusion criteria. See Tables 1, 2 for database-specific search strings and detailed yields.

The databases we searched identified 14,577 records. We removed 11,097 duplicates and screened the remaining 3,480 titles and abstracts. A total of 209 full-text articles were assessed for eligibility, resulting in 52 studies that met all inclusion criteria for the final synthesis. Reasons for exclusion at the full-text stage included ineligible study design (n=60), irrelevant outcomes (n=40), and incomplete or duplicate data (n=57). The selection process is illustrated in the Preferred Reporting Items for Systematic Reviews and Meta-Analyses (PRISMA) flow diagram (Figure 1) [10].

Study Characteristics

The general methodological characteristics and clinical themes of these 52 included studies, with publication dates ranging from 2019 to 2025, are summarized in Table 3. 

**Table 3:** Summary of characteristics of the included studies (N=52) This table provides an aggregate synthesis of the 52 studies included in the scoping review, categorized by methodological design, level of evidence, and primary clinical focus. Study designs were grouped by methodological similarity: "Observational" includes prospective and retrospective cohorts, matched retrospective studies, chart reviews, and machine learning/cluster analyses. "Quality Improvement" encompasses feasibility trials, implementation science studies, and mixed-methods evaluations. "Other" includes narrative reviews, expert commentaries, and bench research animal models. Primary themes were assigned based on the central intervention or research objective of each study. SR/MA: Systematic Review and Meta-Analysis; RCTs: Randomized Controlled Trials; ML/LCCA: Machine Learning/Latent Class Cluster Analysis; LoE: Level of Evidence; N: total number of studies; n: subset number.

Characteristic	Categories	Number of studies (n=52)
Study Design	Systematic Reviews / Meta-Analyses	4
	Randomized Controlled Trials (RCTs)	6
	Observational (Cohort, Case-Control, ML/LCCA, Prospective Observational)	28
	Quality Improvement	8
	Other (Reviews, animal models)	6
Level of Evidence	Level 1 (a/b)	9
	Level 2 (b)	21
	Level 3–5	22
Primary Theme	Remote Monitoring / Telehealth	20
	Pharmacological Protocols	9
	Health Systems, Risk & Education	23

Our review included 52 final studies with publication dates ranging from 2019 to 2025 met all the inclusion criteria for this scoping review. The literature represents a rapidly evolving field, with a marked surge in research output focused on digital health and remote monitoring interventions. 

Geographic and Methodological Diversity

The evidence base for this review has diverse geographic settings and healthcare systems, including the United States, United Kingdom [11], Canada [1], Ghana [12], India [13], and South Korea [14]. Three large-scale systematic reviews and meta-analyses provided the framework for current standardized care, synthesizing data from over 40,000 global participants [7,15,16]. Additionally, six randomized controlled trials (RCTs) evaluated pharmacological comparisons, such as amlodipine versus labetalol, and physician-guided titration models [13,17-21]. Critical risk factors and real world outcomes for readmission were mapped through 34 cohort and case-control studies. We also looked at three qualitative or feasibility studies that explored patient experiences with self-management and the impact of interprofessional telehealth consults [4,11,22].

Participant Characteristics and Settings 

Across the 52 studies we reviewed, the participants were largely patients with known HDP, such as gestational hypertension and preeclampsia. However, we found a critical subset: 20% of those who suffered severe postpartum spikes had no history of high blood pressure during their pregnancy [13]. For these individuals, their condition was entirely new-onset, meaning they wouldn't have been flagged as high-risk before delivery. Study populations ranged from small, specialized pilot groups (n=45) to massive population-level datasets including up to 1.3 million pregnancies.

High-risk profiles within these populations frequently included individuals with obesity (body mass index (BMI) ≥30), Black race, and advanced maternal age (≥40), all of which were consistently identified as predictors for severe complications. Research was conducted across various environments, including tertiary academic hospitals, community safety-net systems, and remote digital health platforms.

Thematic Findings

Theme 1 - Inconsistent risk identification and preventive action: Risk stratification remained significantly inconsistent during the critical transition from hospital discharge to early postpartum follow-up. Recent data using latent class cluster analysis (LCCA) uncovered a specific 'high-risk' group. This cluster represented 31.2% of the population, and these individuals were five times more likely to still have hypertension three months after delivery. Changes in protocol especially at discharge made a huge difference: maintaining a 72-hour hospital stay and sticking to a 140/90 mmHg discharge limit showed a significant decrease in blood pressure spikes compared to the standard care [23].

Furthermore, while early pregnancy screening for markers such as 24-h urinary protein, Pregnancy-Associated Plasma Protein-A (PAPP-A), β-Human Chorionic Gonadotropin (β-hCG), Placental Growth Factor (PLGF), and serum indicators in early pregnancy remained essential for prediction [23], office-based follow-up alone was insufficient for postpartum surveillance. Home blood pressure monitoring was found to be critical for capturing 'masked' hypertension which affects approximately 21% of patients that standard check-ups miss entirely [24-26].

Theme 2 - Pharmacology, adherence, and symptom recognition: Ensuring a patient’s blood pressure is stable for at least 12 hours before they head home is a vital safety step; failing to treat a spike in that final window can increase the chance of readmission by 32%, or even three-fold for those already on medication [27]. To reach this level of stability more effectively, oral amlodipine has proven to be a superior choice over labetalol, bringing blood pressure under control 7.2 hours faster for women dealing with new or persistent postpartum hypertension However, providers should expect that the patients who were started on amlodipine may require longer term use at discharge [13]. Alternative regimens, such as combinations of hydrochlorothiazide and lisinopril, showed an 85% probability of superior short-term control over the standard nifedipine [18].

While immediate postpartum diuretics, including furosemide or torsemide, may lower systolic blood pressure, meta-analyses suggest they did not significantly reduce overall readmission rates or the need for additional antihypertensives [26,28]. Furthermore, patients facing self-management of postnatal hypertension (SNAP-HT), which utilizes telemonitoring and self-adjustment, improved patients' sense of control and reduced their anxiety, directly acknowledging that for most patients, self-care is often the first thing to overlook when balancing newborn care and personal recovery [11,29].

Theme 3 - Health system failures and care coordination: The traditional postpartum follow-up model represented a significant systemic failure by failing to account for high clinical volatility in the immediate weeks after discharge. Initial feasibility data illustrated this "blind spot," revealing that 19% of the obstetric population required specialized hypertensive management; among those monitored in a pilot cohort, 53% required medication adjustments and 16% experienced severe hypertension, events that traditional six-week checkups are chronologically designed too late to capture [4]. In response, integrated coordination models like Heart Safe Motherhood proved superior to standard office-based care, utilizing scalable, low-cost technology to ensure consistent blood pressure ascertainment across diverse practice settings [4,6]. Specialized postpartum transition clinics further strengthened this remote clinical oversight.

Five-year clinical data showed that diverse populations were highly receptive to this model, often attending multiple visits for blood pressure management and CVD prevention [30-32]. These models provided a sustainable framework for capturing high-risk patients who might otherwise be lost to follow-up, such as those in high-distress neighborhoods where traditional barriers to care often led to higher readmission rates [5]. Finally, ensuring that patients with blood pressure ≥140/90 mmHg are provided with antihypertensive medications at discharge further strengthened this safety net, significantly reducing the risk of readmission and severe preeclampsia complications [33].

Theme 4 - Disparities in access and outcomes: Neighborhood and racial disparities significantly drove inequitable postpartum outcomes, creating a "double burden" for high-risk patients [34]. Research indicated that individuals in the most disadvantaged neighborhoods were twice as likely to develop stage 2 hypertension by 21 days postpartum, while Black patients were three to four times more likely to experience these severe spikes by six weeks [35,36]. This vulnerability is historically worsened by the "insurance cliff," where public coverage often expires just 60 days after delivery, leaving a dangerous gap in care for managing hypertension that frequently persisted into the third month postpartum [37,38]. These risks were compounded by weight management; individuals who did not lose weight in the early postpartum period faced four to seven times higher odds of readmission for hypertension or heart failure [39]. Intervention during early postpartum was proven to be a crucial window for identifying the most effective surveillance methods, such as continuous versus intermittent blood pressure monitoring, for individuals with severe features of preeclampsia [40,41].

To meaningfully impact these rates of CVD, the health system must shift toward structured "fourth trimester" follow-ups that prioritize lifestyle interventions. Specifically, focus should be placed on active weight loss and blood pressure reduction strategies, which have been shown to improve both future pregnancy outcomes and long-term heart health [42]. Ultimately, telemedicine serves as a vital tool to facilitate this high-quality care, bypassing traditional physical barriers to ensure that these essential lifestyle and medical interventions reach the populations that need them most [43,44].

The complete data extraction and study characteristics for all 52 articles included in this scoping review are detailed in Table 4.

**Table 4:** Comprehensive summary of all studies included in the scoping review (N=52) ASA: Aspirin; HDP: Hypertensive disorders of pregnancy; ED: Emergency Department; BMI: Body Mass Index; beta-hCG: Beta-human chorionic gonadotropin; PLGF: Placental growth factor; PAPP-A: Pregnancy-associated plasma protein A; hsCRP: High-sensitivity C-reactive protein; PE: Preeclampsia; BP/SBP/DBP: Blood pressure/ Systolic blood pressure/Diastolic blood pressure; CpG: Cytosine-phosphate-Guanine (DNA methylation sites); n: Sample size; LoE: Level of evidence; SR/MA: Systematic Review/Meta-Analysis; Retros.: Retrospective; RCT: Randomized Controlled Trial; QI: Quality Improvement; ML/LCCA: Machine Learning/Latent Class Cluster Analysis; AUC: Area Under the Curve; aRR/RR: Adjusted Relative Risk/Relative Risk; PROBE: Prospective Randomized Open-Blinded End-point; RPM: Remote patient monitoring; HBPM/ABPM: Home blood pressure monitoring/Ambulatory blood pressure monitoring; EMR: Electronic Medical Record; ADI: Area Deprivation Index; ER: Extended Release (e.g., Nifedipine ER); SNAP-HT: Self-Management of Postnatal Anti-hypertensive Treatment; STAMPP-HTN: Systematic Treatment and Management of Postpartum Hypertension; +: Indicates a sample size exceeding the reported value.

Publication Year	Author (Year)	n	Study design	LoE	Key management and outcome
2019	Duley et al. [16]	40,249	SR / MA	1a	Low-dose ASA (50–150 mg) reduced preeclampsia by 18% and neonatal death by 14%, with a marginal increase in postpartum hemorrhage and placental abruption.
2022	Hauspurg et al. [8]	N/A	Review	5	Proposed standardized postpartum HDP criteria emphasizing early antihypertensives, , and diuresis, noting peak symptom onset at 7–10 days.
2024	Tao et al. [23]	413	Retros. cohort	2b	Risk-clustering models found that a ≥140/90 mmHg discharge threshold and ≥3-day hospital stay significantly reduced postpartum blood pressure spikes in high-risk patients.
2025	Chandrasekaran et al. [28]	258	Prospective Cohort	2b	Routine 5-day oral furosemide for gestational hypertension and preeclampsia did not significantly reduce readmission rates or improve one-week blood pressure control, although lower diastolic pressures were observed at six weeks
2025	Kitt et al. [19]	220	PROBE Trial	1b	Postpartum blood pressure self-monitoring paired with remote physician-guided titration significantly lowered aortic diastolic pressure and improved central arterial stiffness at nine months compared to standard primary care.
2025	Sinnott et al. [45]	20,410.	Retros. cohort	2b	Longitudinal EMR analysis revealed that nearly 20% of previously normotensive patients develop blood pressure abnormalities by six months postpartum, highlighting critical gaps in long-term surveillance and the transition to primary care.
2025	Rosenfeld et al. [46]	705	Matched cohort	2b	Remote patient monitoring using a tighter treatment threshold ≥130/80 mmHg for antihypertensive titration significantly reduced postpartum ED visits for hypertensive disorders by 68% and improved blood pressure control at six weeks.
2025	Zullo et al. [7]	714	SR / MA	1a	Remote blood pressure monitoring demonstrated significantly higher emergency department visit rates (9.0% vs. 4.4%) compared to standard care, though hospital readmission rates for hypertensive disorders remained similar.
2024	Lemon et al. [5]	12,038	Matched Retros.	2b	Remote nursing-led management using home blood pressure monitoring significantly reduced six-week readmissions (aRR 0.78) and improved postpartum visit attendance and medication initiation.
2022	Kim et al. [47]	24	Case-control	3b	Placental epigenetic analysis identified significant cytosine (CpG) methylation dysregulation, with a five-marker model achieving high predictive accuracy (AUC 0.99, 95% sensitivity) for new-onset postpartum preeclampsia.
2022	Lovgren et al. [27]	3,480	Retros. cohort	2b	Longitudinal EMR analysis demonstrated that hypertension within 12 hours of hospital discharge increases readmission risk by 32%, suggesting that blood pressure should be normalized for at least 12 hours prior to discharge.
2023	Lemon et al. [35]	4,193	Retros. cohort	2b	Disadvantaged neighborhoods (high Area Deprivation Index) were significantly associated with persistent Stage 2 hypertension at three and six weeks postpartum, although neighborhood factors did not fully account for observed racial disparities in sustained high blood pressure.
2025	Amar et al. [1]	1,317,181	Retros. cohort	2b	Population-level analysis found that new-onset postpartum preeclampsia significantly increases the risk of severe maternal morbidity (RR 6.48), placental abruption, and recurrence in subsequent pregnancies (7.77-fold increase).
2021	Suresh et al. [2]	926	QI / prospective	3	Implementation of a hospital-wide clinical bundle—including standardized education and protocols—significantly increased postpartum follow-up adherence from 33.5% to 59.4% and improved blood pressure control consistently across all racial groups.
2023	Parker et al. [48]	2,465	Retros. cohort	2b	Retrospective analysis identified a 12.1% incidence of de novo postpartum hypertension, with non-Hispanic Black patients and those over age 35 at highest risk, and 22% of cases diagnosed after the standard 6-week visit.
2019	Hoppe et al. [4]	55	Feasibility	4	A nurse-led telehealth program achieved 95% retention and zero hospital readmissions, even though 53% of participants required medication adjustments for blood pressure exacerbations.
2024	Bisson et al. [49]	1,480	Retros. study	2b	Evaluation of the STAMPP program found that only 63% of patients had follow-up within one year, with those lacking care more likely to have public insurance and Black patients showing disproportionately low rates of primary care and cardiology follow-up.
2025	Patel et al. [36]	306	Prospective cohort study	2b	A routine remote monitoring program achieved high initial attendance (84%), but Black patients experienced significantly higher rates of Stage 2 hypertension at six weeks (22.4% vs. 2.2%) and greater engagement attrition over time.
2020	Triebwasser et al. [6]	436	Implementation	3	Hospital-wide implementation of a text-based remote monitoring program achieved 95.5% blood pressure ascertainment and ensured 84.7% of patients met clinical guidelines regardless of race.
2022	Hacker et al. [50]	5,959	Prospective Obs	2b	A universal patient-driven home blood pressure monitoring program for previously normotensive women proved feasible and identified an 8% rate of new hypertensive disorders and a 0.7% rate of severe hypertension.
2024	Huynh [29]	220	Research highlight	5	Postpartum blood pressure self-monitoring with a physician-led titration algorithm significantly lowered diastolic blood pressure and reduced left ventricular mass index at nine months compared to usual care.
2025	Woolcock Martinez et al. [40]	25	Pilot Comp	4	Continuous 24-hour finger plethysmography was significantly more sensitive than standard intermittent monitoring, detecting severe-range systolic hypertension in 44% of participants compared to only 8% via oscillometric checks.
2025	Pratt et al. [20]	132	Pragmatic RCT	1b	Oral amlodipine was found to be non-inferior to nifedipine ER regarding postpartum length of stay, with both medications demonstrating similar efficacy and safety profiles for managing postpartum hypertension.
2023	Hayden-Robinson et al. [51]	185	QI / Retros.	3	A home blood pressure monitoring program with standardized self-triage guidance identified postpartum elevations in 20% of patients, leading to medication adjustments for 28% and early obstetric triage for 9% of the cohort.
2020	Wen et al. [41]	65,401	Retros. cohort	2b	Evaluation of 60-day hypertension-related readmission risk found that longer hospital stays (5–7 days and >7 days) significantly decreased readmission risk compared to stays under 3 days, with 90% of readmissions occurring within 10 days of discharge.
2025	Roman et al. [32]	157	Retros. chart review	3	Implementation of a specialized postpartum hypertension clinic for 72-hour follow-up resulted in 53% of patients meeting the follow-up goal, with 58% achieving target blood pressure at subsequent visits and a 5% readmission rate.
2024	Lemon et al. [39]	1,365	Retros. cohort	2b	Analysis of early postpartum weight change found that failure to lose weight within 10 days of delivery was associated with four to seven times higher odds of readmission and adverse blood pressure trajectories, serving as an accessible marker for identifying high-risk individuals.
2022	Sanghavi et al. [44]	236	Retros. cohort	3	Comparison of follow-up strategies found that telemedicine significantly increased visit completion (70% vs. 32%) compared to in-person care and helped mitigate racial and age-based disparities in postpartum hypertension management.
2024	Safri et al. [22]	45	QI / Pharmacy	4	A pharmacist-led e-consult model using remote monitoring and a collaborative practice agreement enabled prompt medication initiation (54 prescriptions) and timely, equitable care for a high-risk, diverse postpartum population.
2024	Moustafa et al. [52]	250	Prospective cohort	2b	A program combining standardized education with six weeks of remote monitoring significantly improved patient knowledge, but neither knowledge nor engagement was associated with improved visit attendance, with maternal age acting as the primary driver of study participation.
2023	Fishel Bartal et al. [18]	70	Pilot RCT	1b	A trial comparing antihypertensives found that combined oral hydrochlorothiazide and lisinopril had an 85% probability of superior blood pressure control (lower rates of Stage 2 hypertension) on days 7–10 postpartum compared to nifedipine.
2022	Nuckols et al. [26]	62	Prospective comp	2b	Assessment of 1–4 year postpartum monitoring showed that 7-day home blood pressure monitoring (HBPM) has excellent diagnostic agreement with 24-hour ambulatory monitoring (ABPM) and successfully identified 21% of masked hypertension cases missed by in-office readings.
2018	Viteri et al. [21]	118	Double-blind RCT	1b	A randomized controlled trial found that oral torsemide (20 mg/day) for five days did not significantly reduce rates of persistent postpartum hypertension (44% vs. 58%) or hospital readmission compared to a placebo.
2023	Christenson et al. [53]	82	Prospective obs	2b	Analysis of biomarkers and clinical factors revealed that prenatal low-dose aspirin use was associated with a 0% incidence of postpartum hypertension and lower antiangiogenic factor levels, significantly reducing six-week re-hospitalization rates.
2023	Aderibigbe et al. [17]	256	Pragmatic RCT	1b	A trial comparing blood pressure thresholds for antihypertensive initiation found no significant difference in composite maternal morbidity (8.6% vs. 11.7%) or hospital readmission between "tight control" (≥ 140/90 mmHg) and "liberal control" (≥ 150/95 mmHg).
2021	Kern-Goldberger et al. [43]	N/A	Review	5	Telemedicine and text-based monitoring are identified as critical tools for delivering high-quality, equitable care, effectively reducing racial disparities by overcoming physical and systemic barriers to traditional in-person postpartum follow-up.
2021	Smithson et al. [54]	99	Retros. cohort	2b	Evaluation of de novo postpartum hypertension risk factors (in patients with no prior history of HDP) identified maternal age ≥40, BMI ≥30, Black race, and antenatal low-dose aspirin use as significant predictors of hospital re-presentation within 30 days.
2025	Wall et al. [37]	N/A	QI Report	5	The "Hypertension in Pregnancy Change Package" provides a standardized framework for outpatient clinicians to adopt lower blood pressure thresholds for treatment, aimed at reducing maternal and neonatal complications through evidence-based care processes and improved transition of care.
2022	Suresh et al. [34]	N/A	QI	5	This quality improvement framework utilizes standardized clinical protocols and self-measured blood pressure monitoring to enhance the timely detection of hypertension and significantly reduce maternal morbidity throughout the postpartum period.
2019	Celi et al. [30]	412	QI / Retros	3	Implementation of a specialized "Postpartum Transition Clinic" resulted in 48.3% of patients requiring medication adjustments, while increasing home monitor acquisition to 93.8% and achieving a 79.5% successful transition rate to primary care.
2023	Gupta et al. [13]	130	RCT	1b	A comparative study found that oral amlodipine achieved sustained blood pressure control 7.2 hours faster than oral labetalol and resulted in fewer severe hypertensive episodes, though amlodipine users were more likely to require continued medication at discharge.
2022	Fondjo et al. [12]	130	Case-control	3b	An evaluation of new-onset versus persistent postpartum preeclampsia identified shared risk factors including physical inactivity, infrequent antenatal visits, analgesic use, and cesarean delivery, while contraceptive use was a unique predictor for new-onset cases.
2022	Li et al. [24]	1,000	ML / LCCA	3	Application of Latent Class Cluster Analysis (LCCA) identified three distinct risk clusters for persistent postpartum hypertension, with the high-risk cluster (31.2% of the cohort) being five times more likely to remain hypertensive at three months postpartum compared to low-risk groups.
2019	Hauspurg et al. [42]	315	Prospective cohort	2b	Characterization of the cohort seven months postpartum revealed that 29% had sustained hypertension. Preeclampsia was associated with a 2.35-fold increased risk of sustained hypertension and a 3.23-fold increased risk when coupled with elevated cardiometabolic biomarkers (Cystatin C and hsCRP) compared to normotensive pregnancies.
2024	Ye et al. [25]	186	Case-control	3b	Evaluation of biochemical and placental markers showed that patients with preeclampsia (PE) had significantly higher urinary protein and β-hCG levels, alongside lower PLGF and PAPP-A; combined screening in early pregnancy effectively predicts PE risk and associated adverse outcomes.
2025	Keen et al. [15]	1,273	SR / MA	1a	Evaluation of immediate postpartum diuretic treatment for hypertensive disorders of pregnancy (HDP) found that while diuretic use was associated with significantly lower systolic blood pressure, it did not significantly reduce diastolic blood pressure, rates of persistent hypertension, or the need for additional antihypertensive medications.
2025	Pihelgas et al. [31]	470	Retros case-control	3b	Evaluation of a specialized "Postpartum Preeclampsia Clinic" showed that attendance was associated with lower rates of postpartum weight gain, a lower incidence of subsequent hypertension, and significantly higher rates of successful transition to primary care.
2021	Li et al. [55]	Rats	Animal model	5	Prophylactic administration of magnesium sulfate () in a lipopolysaccharide-induced preeclampsia model significantly reduced postpartum hypertension and organ inflammation; this was achieved by promoting a shift from pro-inflammatory (M1) to anti-inflammatory (M2) macrophage polarization in both the kidney and the brain.
2020	Sawyer et al. [38]	N/A	Commentary	5	Rapid implementation of remote blood pressure monitoring (telehealth and home BP cuffs) was found to be feasible and significantly improved compliance with ACOG guidelines. The model increased six-week postpartum visit attendance from 66% to 88% and effectively reduced racial disparities in care engagement.
2022	Kim et al. [14]	209	Retros. cohort	2b	A comparison of treatment strategies found that continuous nicardipine infusion resulted in significantly lower blood pressure levels starting 16–20 hours postpartum and reduced the cumulative requirement for additional antihypertensive agents compared to consecutive bolus therapy with labetalol or hydralazine.
2020	Cairns et al. [11]	68	Mixed methods	3	Evaluation of the "SNAP-HT" intervention showed that self-management via daily home monitoring and automated medication down-titration significantly enhanced patient autonomy and reduced blood pressure-related anxiety, allowing for more reactive and targeted treatment adjustments compared to usual care.
2023	Wei [33]	11,260	Retros cohort	2b	Implementation of a text-based remote blood pressure monitoring program was associated with a significant reduction in seven-day hospital readmissions for hypertension (0.7%) compared to standard office-based care (1.1%).

Discussion

This scoping review mapped current postpartum follow-up and management strategies which identified that the severe maternal outcomes persist primarily due to the gaps of care and the failure of maintaining continuity of care. While standardized protocols like the "Hypertension in Pregnancy Change Package" exist, clinician recognition of risk remains variable. Evidence indicates that relying on traditional office visits alone is insufficient; while 84.0% of patients can successfully utilize RPM [4,37], office-based follow-up attendance is often as low as 30-50%. Furthermore, while medications like oral amlodipine achieve faster blood pressure control than labetalol, the lack of standardized discharge thresholds, such as the 140/90 mmHg limit, often leads to preventable postpartum spikes [13].

Interpretation

The identified gaps are driven by a combination of structural and clinical factors, with the disconnect between inpatient and primary care serving as the single biggest point of failure. Nearly 20% of patients who were normotensive at delivery exhibited blood pressure abnormalities within six weeks, yet the transition to primary care was often incomplete. While current guidelines recommend a primary care provider (PCP) visit within the first year for individuals with a history of HDP, this transition was rarely successful. This gap is a critical missed opportunity for intervention, as CVD remains the leading cause of mortality among these patients, and a diagnosis of preeclampsia effectively doubles their long-term risk for CVD [49].

This disconnect was further magnified by "masked" hypertension, where in-office readings appeared normal despite high home readings in 21% of cases. Additionally, more than one in 10 patients with normotensive pregnancies experienced de novo postpartum hypertension in the year following delivery [48]. By six months postpartum, nearly one-fifth of a large, previously normotensive cohort had developed BP abnormalities per American College of Cardiology-American Heart Association criteria [3,45]. These gaps were not evenly distributed; Black race and high neighborhood deprivation (Area Deprivation Index) remained independent predictors of persistent hypertension. In the United States, the maternal mortality rate for Black women remains approximately 3.5 times higher than for white women (50.3 vs 14.9 deaths per 100,000 live births).

To specifically address these geographic inequities, the implementation of free mobile health clinics dedicated to postpartum surveillance offers a vital mechanism to bypass transportation barriers and bring essential monitoring directly into underserved communities. When RPM is woven into broader hospital-wide quality initiatives, it effectively keeps patients connected to their care teams during the vulnerable weeks after birth. For those actively using these digital tools, there is a clear trend of stabilizing blood pressure levels over a six-week period. However, the data also revealed a persistent challenge: significant racial imbalances still existed in both the severity of hypertension and the frequency with which patients engage with monitoring technology. Addressing these inequities remains a critical hurdle for future clinical strategies [37].

In the past, these health divides were often worsened by the "60-day cliff," where insurance coverage would abruptly end just two months after delivery. The current shift toward widespread 12-month Medicaid extensions has created a vital opening for Family Medicine physicians. This extended coverage provides a much-needed window to move beyond immediate crisis management and focus on reducing a patient's lifelong cardiovascular risk [37].

Clinical Implication

To effectively manage postpartum blood pressure and prevent the risk of severe hypertensive-related complications, clinicians should implement a standardized set of interventions that have been proven to protect patients during the transition to home care. According to the ACOG, the management of postpartum hypertensive disorders centers on the acute stabilization of severe-range blood pressures (≥160/110 mmHg) to mitigate the immediate risk of cerebrovascular accidents. This foundational protocol necessitates the use of rapid-acting antihypertensives, such as IV labetalol, IV hydralazine, or oral immediate-release nifedipine, alongside magnesium sulfate (MgSO₄) for seizure prophylaxis in patients with severe features [3]. Furthermore, ACOG guidelines mandate a structured follow-up evaluation within three to 10 days post-discharge, with an accelerated 72-hour window for those diagnosed with severe features, to ensure the detection of secondary blood pressure peaks [3]. Regarding pharmacological optimization, amlodipine is more efficient for rapid stabilization for immediate control, while MgSO₄ remains the gold standard for seizure prophylaxis, potentially working through anti-inflammatory macrophage polarization [55]. 

Beyond pharmacological treatment, the structure of the follow-up system itself is a vital clinical tool. Specialized multidisciplinary clinics, such as Postpartum Transition or Vascular Risk Reduction clinics, effectively improve long-term primary care follow-up to 79.5%. These clinics provide more than just monitoring; they help patients manage postpartum weight, which is a key factor in reducing future cardiovascular risks [31]. Furthermore, the integration of free mobile health units could serve as a 'bridge' for patients who are unable to reach physical clinic locations, ensuring that high-risk monitoring and follow-up care are not interrupted by financial or logistical constraints.

Given that roughly 60% of maternal deaths in the United States occur after the patient has left the hospital, there is an urgent need for a more robust safety net [52]. Establishing universal home blood pressure monitoring programs allows for closer surveillance of all women, especially those with existing risk factors or those living in areas with limited medical resources [50,54]. Quality improvement initiatives, such as Systematic Treatment And Management of PostPartum Hypertension (STAMPP HTN), have been instrumental in identifying exactly where care breaks down between delivery and the first postpartum checkup [52]. By providing every patient who has a hypertensive diagnosis with a home monitor, we can better identify rising blood pressure early and step in before a preventable adverse event occurs [51].

Fifteen high-impact studies, summarized in Table 5, highlight the most clinically actionable data.

**Table 5:** Summary of the clinical characteristics and primary findings of representative studies (N=15) Studies were selected based on their Level of Evidence (LoE) and direct relevance to postpartum hypertension management and eclampsia prevention. ↑/↓: Increased/Decreased; +: Indicates a sample size exceeding the reported value; aRR/RR: Adjusted relative risk/Relative risk; ASA: Acetylsalicylic acid (Aspirin); BP/SBP/DBP: Blood pressure/Systolic blood pressure/Diastolic blood pressure; CI: Confidence interval; ED: Emergency department; HDP/HTN/PE: Hypertensive disorders of pregnancy/Hypertension/Preeclampsia; LoE: Level of evidence; n: Sample size; PROBE: Prospective randomized open blinded end-point; pts: Patients; QI: Quality improvement; RCT: Randomized controlled trial; Retros.: Retrospective; RPM: Remote patient monitoring; SR/MA: Systematic review and meta-analysis; tx: Treatment.

Study (Year)	Design	LoE	n	Primary Focus	Key Findings (95% CI / p-value)
Amar et al. (2025) [1]	Retros. Cohort	2b	1.3M+	New-onset PE Risk	↑ Morbidity (RR 6.48) and 7.7x ↑ recurrence risk.
Wen et al. (2020) [41]	Retros. Cohort	2b	65,401	Readmission Timing	90% of readmissions occur within 10 days of discharge.
Duley et al. (2019) [16]	SR / MA	1a	40,249	Prevention (ASA)	↓ Preeclampsia (PE) 18%; ↓ neonatal death 14%.
Sinnott et al. (2025) [45]	Retros. Cohort	2b	20,410	Long-term Risk	20% of normotensive pts develop HTN within 6 months.
Lemon et al. (2024) [5]	Matched Retros.	2b	12,038	RPM (Nursing)	↓ 6-wk readmissions (aRR 0.78); ↑ visit attendance.
Wei (2023) [33]	Retros. Cohort	2b	11,260	RPM (Text-based)	↓ 7-day readmissions (0.7% vs. 1.1%; p<0.05)
Parker et al. (2023) [48]	Retros. Cohort	2b	2,465	De novo HDP	12.1% incidence; age >35 & Black race as top predictors.
Keen et al. (2025) [15]	SR / MA	1a	1,273	Loop Diuretics	↓ SBP; no effect on DBP or persistent hypertension.
Suresh et al. (2021) [2]	QI / Prospective	3	926	Care Equity	↑ Follow-up (33% to 59%); eliminated racial gap in control.
Zullo et al. (2025) [7]	SR / MA	1a	714	RPM Safety	↑ ED visit rate (9% vs 4%); hospital readmission similar.
Rosenfeld et al. (2025) [46]	Matched Cohort	2b	705	Tight Control	↓ ED visits by 68% using ≥130/80 mmHg threshold.
Triebwasser et al. (2020) [6]	Implementation	3	436	RPM Feasibility	95.5% BP ascertainment; 84.7% guideline compliance.
Kitt et al. (2025) [19]	PROBE Trial	1b	220	Titration Logic	↓ Aortic DBP and arterial stiffness at 9 months.
Pratt et al. (2025) [20]	Pragmatic RCT	1b	132	Amlodipine	Non-inferior to Nifedipine ER; similar efficacy/safety.
Hauspurg et al. (2022) [8]	Review	5	N/A	Guidelines	Peak symptom onset 7–10 days; standardized tx protocols.

Research Implications

Future research must prioritize implementation-focused studies on scalable RPM programs. Specifically, we should explore using machine learning tools like LCCA to help us pinpoint that critical 30% of patients who are at the highest risk and truly need the most intensive oversight [24]. Future studies should also evaluate the comparative effectiveness of mobile health clinics versus traditional office-based models in improving visit adherence and longitudinal blood pressure control among low-income and rural populations. Studies should also investigate the long-term impacts of epigenetic Cytosine-phosphate-Guanine (CpG) markers and the long-term benefits of prenatal aspirin. In some early observational groups, aspirin use was actually associated with a 0% rate of postpartum hypertension, a lead that definitely deserves more attention [47,53]. 

Furthermore, we’ve seen that when patients used remote monitoring to keep their blood pressure under 130/80 mmHg and received their prescriptions before they even left the hospital, they were significantly less likely to end up back in the emergency room for hypertensive issues. The next big question for researchers is whether adopting these stricter targets and moving beyond the current ACOG-recommended discharge stability threshold of <160/110 mmHg for 24 hours, can fundamentally change the long-term outlook for these patients, lower readmission rates, and potentially prevent the development of postpartum preeclampsia [3,46,54].

Limitations

The included studies were heterogeneous in design, ranging from large-scale retrospective analyses (n=11,260 to 1.3 million) to small pilot RCTs (n=45 to 62). There was a lack of long-term (one to five years) follow-up data in many telehealth-focused studies. Additionally, the concentration of research in high-resource settings raises questions about the generalizability of these remote monitoring strategies to lower-resource areas. The lack of a unified clinical definition for postpartum hypertension across these studies further creates a 'standardization gap,' making it a challenge to pinpoint a consistent baseline for successful treatment.

## Conclusions

The transition from hospital to home is the most dangerous "missed opportunity" in maternal health. While pharmacological agents like amlodipine and nicardipine provide robust blood pressure control, their clinical efficacy is entirely relied upon a system that can monitor the patient after they leave the hospital. This review identifies that the traditional office-based follow-up model is insufficient, failing to capture both de novo hypertension and the "masked" spikes that occur in nearly a quarter of cases.

To bridge this "transition gap," the evidence supports a shift toward stricter clinical thresholds and technology-enabled surveillance. Crucially, the data suggest that maintaining a stable 12-hour normotensive buffer before discharge can significantly lower readmission risks, especially when combined with tighter blood pressure targets, such as keeping readings below 130/80 mmHg. The most effective strategies identified to reduce readmissions and address racial disparities in follow-up were utilizing text-based RPM, specialized postpartum clinics, and the deployment of free mobile health units to eliminate geographic and transportation barriers. Ultimately, by adopting a standardized transition of care from hospital to primary care and utilizing extended 12-month Medicaid coverage, clinicians can transform the "fourth trimester" from a period of high-risk fragmentation into a vital window for life-long cardiovascular health optimization.
